# Simulating the Electronic Circular Dichroism of Chlorophyll b in the Presence of a Gold Nanosphere

**DOI:** 10.1021/acs.jpcb.5c06445

**Published:** 2025-12-16

**Authors:** Rilinda Plakaj, Leonardo Biancorosso, Eleonora Luppi, Emanuele Coccia

**Affiliations:** † Dipartimento di Scienze Chimiche e Farmaceutiche, 9315 Università di Trieste, via L. Giorgieri 1, 34127 Trieste, Italy; ‡ Laboratoire de Chimie Théorique, Sorbonne Université, CNRS, Paris F-75005, France

## Abstract

In this work, we apply a real-time multiscale approach to investigate plasmonic effects arising from a gold spherical nanoparticle (NP) on the electronic circular dichroism (ECD) spectrum of Chlorophyll b (Chlb). Chlb has been described within linear-response time-dependent density functional theory (TDDFT) to obtain excitation energies and transition dipole moments, which were then employed in a TD-CIS ansatz for the time-dependent wave function, subsequently propagated by solving the time-dependent Schrödinger equation. Three different distances (1, 3, and 5 nm) of the molecule with respect to the surface of the NP were considered. For each distance, three Chlb orientations (two perpendicular and one parallel) were taken into account, together with three pulse polarization directions. In addition, a simple descriptor of the plasmon-induced signal enhancement or quenching of the signal was introduced, based on the time-dependent coherences between the electronic ground state and selected excited states. In general, modifications in the ECD spectra of Chlb have been attributed to plasmonic effects. An interesting exception is the appearance of an intense peak at 2.36 eV when Chlb is oriented perpendicularly to the NP surface and the pulse is polarized perpendicular to the molecular plane. In this case, the magnetic transition dipole moment to the second state (providing the excitation at 2.36 eV) increases by a factor of four to five compared to bare Chlb or other orientations. The composition of the excitations was analyzed through the differential projected density of states.

## Graphical abstract

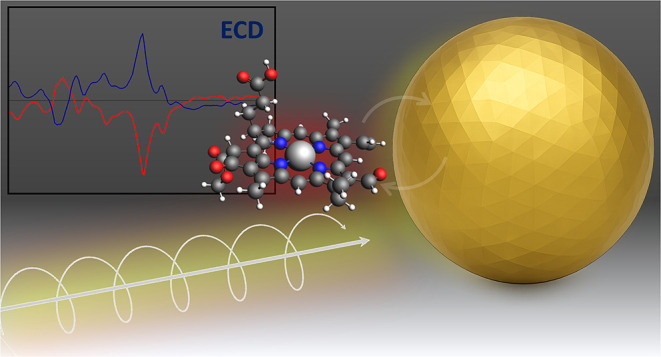

## 1 Introduction

Electronic circular dichroism (ECD) is a fundamental spectroscopic property of chiral molecules.^1–8^ Chiral systems are those whose mirror images cannot be superimposed on the original structure. ECD measures the differential absorption of left- and right-circularly polarized light by enantiomers of chiral molecules. However, a major limitation of ECD is the typically weak signal, which is often challenging to detect.^9^ To overcome this limitation, molecular nanoplasmonics^10,11^ can be considered as a compelling solution, exploring the influence of plasmon-induced processes on the properties of molecular systems near metallic nanoparticles (NPs), allowing enhancement and control of the molecular optical properties through localized surface plasmon resonances (LSPRs).^12–19^

Plasmonic effects are commonly exploited to amplify otherwise weak molecular signals in the presence of NPs, as exemplified by surface-enhanced Raman scattering (SERS).^16,20–24^ Beyond SERS, the optical response of chiral molecules including optical rotation, circular dichroism (CD), and Raman optical activity can also be strongly enhanced through their coupling with LSPRs.^25–28^ Plenty of theoretical work has been dedicated to dipole-based models of the chiral molecule,^27,29–48^ usually formulated within a master equation for the molecular quantum states. Other studies, instead, focus on a full quantum description of the target, which limits the accessible metal size.^49,50^

Another option is provided by time-dependent multiscale hybrid approaches, where the chiral system is fully described quantum mechanically, while the NP is treated classically,^51–54^ using the polarizable continuum model (PCM) coupled to the boundary element method (BEM).^55^ This methodology, referred to as TD-PCM-NP, i.e., time-dependent PCM for NP, was introduced in ref.^56^. This approach has recently been applied to compute the ECD spectra of methyloxirane and peridinin in the presence of a gold nanosphere,^57^ successfully predicting the occurrence or suppression of plasmonic effects depending on the energy gap between the NP plasmonic resonance and the molecular excitations. Since the magnetic response of the NP is not included, only changes in molecular peaks are possible. The ECD spectrum is obtained directly from the response of the time-dependent induced magnetic dipole to an electric field perturbation from a linearly polarized pulse, within linear-response theory.^2^

In this work, we study the ECD of a single Chlorophyll b (Chlb, Figure 1) molecule in the presence of a spherical gold NP, as a function of the Chlb-NP distance, Chlb orientation, and the polarization direction of the pulse. Inspired by the work of ref 58., which demonstrated how plasmonic NPs can enhance the light absorption of Photosystem I, we focus here on the response of a single molecule interacting with a single NP, aiming to elucidate the microscopic mechanisms underlying plasmon-induced modifications in the ECD spectra of Chlb. In addition, we introduce a coherence-based descriptor to qualitatively capture the enhancement or quenching of molecular peaks.

**Figure 1:**
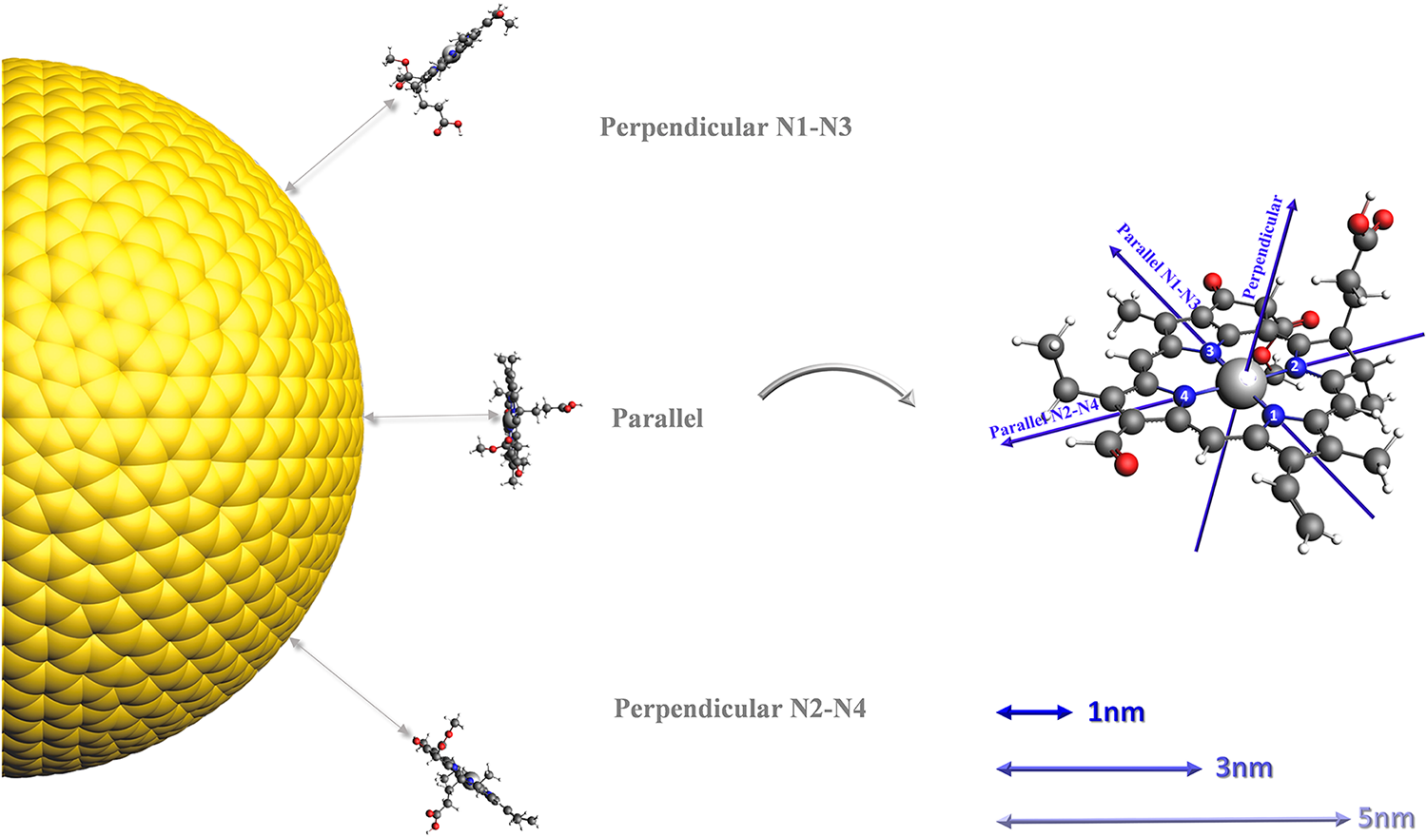
Schematic representation of three relative orientations of Chlb with respect to the NP surface (Parallel, Perp N1–N3 and Perp N2–N4), together with the pulse polarizations adopted in this work.

The article is organized as follows: in Section 2 the theoretical framework is briefly reviewed, with emphasis on the time-dependent formulation of the multiscale problem. Computational details are provided in Section 3, while results are presented and discussed in Section 4. Finally, conclusions are drawn in Section 5.

## 2 Theory

### 2.1 Multiscale Real-Time Propagation

Our multiscale approach couples the NP polarization, described within the PCM, to a quantum representation of the electronic degrees of freedom of the chiral molecule. The theory is formulated in time domain through the time-dependent Schrödinger equation (TDSE), and atomic units are used throughout.

The computational protocol is articulated in three main steps: (i) defining the basis for the time-dependent wave function |Ψ­(*t*)⟩ and propagating the TDSE in the state space in the presence of the NP and the external pulse; (ii) computing the plasmon-modified ECD spectrum;^57^ and (iii) optionally analyzing the photoinduced electron dynamics with postprocessing tools.^59^

Within BEM, the NP surface is discretized into *N*_*T*_ triangular tesserae carrying apparent charges.^55^ The time evolution of these charges captures the NP polarization induced by the external pulse and by the mutual interaction with the time-dependent electronic density of the quantum target.

The TDSE in length gauge for TD-PCM-NP reads as

1 $$i\frac{d}{\mathrm{dt}}\left|\Psi \left(t\right)\right\rangle =Ĥ\left(t\right)\left|\Psi \left(t\right)\right\rangle$$

with *Ĥ*(*t*) being the time-dependent Hamiltonian

2 $$Ĥ\left(t\right)={Ĥ}_{0}-\vec{\mathrm{\mu ̂}}\cdot {\mathrm{E⃗}}_{\mathrm{ext}}\left(t\right)+\left(q_{\mathrm{ref}}\left(t\right)+\Delta q_{\mathrm{pol}}\left(t\right)\right)\cdot {\mathrm{V̂}}_{\mathrm{BEM}}$$

where μ̂⃗ is the molecular dipole operator, *E⃗*_ext_(*t*) is the external laser pulse, **q**_ref_(*t*) are the BEM charges directly induced by the external field, and Δ**q**_pol_(*t*) = **q**_pol_(*t*) – **q**_GS_ represents the time-dependent polarization charges of the nanoparticle (NP), defined as the difference between the instantaneous induced charges **q**_pol_(*t*) and the reference ground-state charges **q**_GS_ in absence of the external field.^51,60^ **V̂**_BEM_ is the molecular electrostatic potential evaluated at the positions of the *N*_*T*_ tesserae on the NP surface. The vectors **q**(*t*), Δ**q**_pol_(*t*) and **V̂**_BEM_ all have dimension *N*_*T*_, i.e., the number of discretization points on the NP surface. These quantities therefore depend on the position of the center of the tessera.

The term *Ĥ*_0_ in eq 2 denotes the field-free electronic Hamiltonian, defined as

3 $${Ĥ}_{0}={Ĥ}_{\mathrm{el}}+q_{\mathrm{GS}}\cdot {\mathrm{V̂}}_{\mathrm{BEM}}$$

where *Ĥ*_el_ is the electronic Hamiltonian of the isolated molecule. Further details can be found in refs. 57,60

In eq 1, |Ψ­(*t*)⟩ is defined as a linear combination of the *N*_states_ eigenstates of the effective field-free Hamiltonian *Ĥ*_0_ of eq 3, which already includes the ground-state polarization of both the molecule and the NP

4 $$\left|\Psi \left(t\right)\right\rangle =\sum_{M=0}^{N_{\mathrm{states}}-1}C_{M}\left(t\right)\left|M\right\rangle$$

where *C*_*M*_(*t*) are time-dependent coefficients, and |*M*⟩ is the *M-*th eigenstate of the system, with eigenvalue *E*_*M*_. To obtain the *H*_0_ eigenstates, the initial guess for the self-consistent calculation is given by using the eigenstates of the electronic Hamiltonian *Ĥ*_el_ of the bare molecule. In practice, the basis set adopted in this work consists of the Kohn–Sham ground state together with the (*N*_states_ – 1) excited states obtained from TDDFT in the singly excited ansatz (TD-CIS),^59,61,62^

5 $$\left|M\right\rangle =\sum_{i}^{\mathrm{occ}}\sum_{a}^{\mathrm{vir}}d_{i,M}^{a}\left|\Phi_{i}^{a}\right\rangle$$

where |Φ_*i*_^*a*^⟩ is the singly excited Slater determinant, constructed by promoting one electron from the occupied orbital *i* to the virtual orbital *a*, and *d*_*i*,*M*_^*a*^ are the corresponding expansion amplitudes.

After the self-consistent step, the set of states |*M*⟩ and the ground-state charges **q**_GS_ provide the reference basis for the time propagation of the TDSE according to eqs 1 and 2. In the absence of the NP, the Hamiltonian reduces to *Ĥ*(*t*) = *Ĥ*_el_ – μ̂⃗ · *E⃗*_ext_(*t*), and the basis for |Ψ­(*t*)⟩ is simply given by the eigenstates of *Ĥ*_el_.

From this point on, we no longer distinguish between the eigenstates of the isolated molecule and those of the molecule interacting with the NP, and we will denote them generically as |*M*⟩ or |*L*⟩.

Explicitly, at the position *R⃗*_*T*_ of each tessera *T* the transition electrostatic potential between two states |*L*⟩ and |*M*⟩ is therefore defined as

6 $${\mathrm{V̂}}_{\mathrm{BEM}}^{L,M}\left({\mathrm{R⃗}}_{T}\right)=\left\langle L\left|\frac{1}{\left|{\mathrm{R⃗}}_{T}-\mathrm{r⃗}\right|}\right|M\right\rangle +{\mathrm{V̂}}_{\mathrm{nuc}}\left({\mathrm{R⃗}}_{T}\right)$$

where **V̂**_nuc._(*R⃗*_*T*_) is the nuclear contribution at position *R⃗*_*T*_.

In order to simulate a kick pulse, the external electric field *E⃗*_ext_ (*t*) is given by a narrow linear Gaussian function

7 $${\mathrm{E⃗}}_{\mathrm{ext}}\left(t\right)=\mathrm{n⃗}{\mathrm{E⃗}}_{\max}t\exp\left(-\frac{{\left(t-t_{0}\right)}^{2}}{2\sigma^{2}}\right)$$

where *t*_0_ is the center and σ the amplitude of the Gaussian function, respectively, *E⃗*_max_ is the maximum amplitude of the field, and *n⃗* is the unit vector defining the polarization axis. The choice of the pulse prevents numerical artifacts possibly affecting the Fourier transform of the time-dependent signal. Indeed, a linear Gaussian pulse guarantees that the pulse is exactly zero at the initial time, thus avoiding truncation of a pure Gaussian tail that, even if the values are very small, could affect the Fourier transform.

### 2.2 Computing the ECD Spectrum

The magnetic dipole moment operator *m̂⃗* is defined neglecting the spin contribution, as

8 $$\mathrm{m⃗}=-\frac{1}{2c}\mathrm{r⃗}\times \mathrm{p⃗}$$

with *r⃗* and *p⃗* being the position and momentum operators, respectively, and *c* being the light speed.

The induced magnetic dipole Δ*m⃗*(*t*) is defined as the difference between the time-dependent magnetic moment *m⃗*(*t*) at time *t* and that at initial time (*t* = 0), *m⃗*(0). The magnetic moment at time *t* is given by

$$\mathrm{m⃗}\left(t\right)=\underset{L,M}{\sum }C_{L}^{*}\left(t\right)C_{M}\left(t\right)\left\langle L\right|\overset{⃗}{\mathrm{m̂}}\left|M\right\rangle ,$$ 9

with ⟨*L*|*m̂⃗*|*M*⟩ explicitly computed at TDDFT level according to eq 25 of ref 57.

The ECD spectrum is computed as the imaginary part of the following expression:^8,63,64^

$$P_{\alpha \beta }^{\mathrm{ECD}}\left(\omega \right)=-\frac{i}{2\pi \omega {\mathrm{E⃗}}_{\mathrm{ext},\alpha }\left(\omega \right)}\int_{0}^{+\infty }-\Delta {\mathrm{m⃗}}_{\beta }\left(t\right)e^{i\left(\omega +i\Gamma \right)t}dt$$ 10

In eq 10, *E⃗*_ext,α_(ω) is the Fourier transform of the α-th Cartesian component of the electric field of the external pulse, Δ*m⃗*_β_(*t*) is the β-th Cartesian component of the induced magnetic dipole (*m⃗*(*t*) – *m⃗*(0)), and Γ is a damping parameter.^63,65^ In this work, we have only considered the signal detection along the polarization axis.

### 2.3 Coherence Descriptor

We define a coherence descriptor to connect the discussion of the enhancement or quenching of the CD signal as a function of the distance between Chlb and the NP with the underlying photoinduced dynamics. Since the magnetic transition dipole moment matrix is antisymmetric, only coherence terms of the type *C*_L_^*^(*t*)*C*_*M*_(*t*) contribute to the time-dependent magnetic dipole moment in eq 9. To rationalize this descriptor, let us consider a transition from the ground state |0⟩ to the state |*M*⟩. The corresponding magnetic dipole α-component at time *t* is

11 m→α(t)=C0*(t)CM(t)⟨0|m̂α|M⟩+CM*(t)C0(t)⟨M|m̂α|0⟩=2iIm[C0*(t)CM(t)]⟨0|m̂α|M⟩.

where α labels the Cartesian component.

The coherence descriptor *C̃*_*M*_ is finally defined as

12 $${\mathrm{C̃}}_{M}=\frac{{\mathrm{C̅}}_{M}^{\mathrm{NPX}}}{{\mathrm{C̅}}_{M}^{\mathrm{bare}}}$$

where the superscripts “NPX” or “bare” indicate, respectively, the time-averaged absolute coherence value when the NP is located at a distance *X* nm from Chlb, or in the absence of the NP (bare Chlb). Formally, the time-averaged *C̅*_*M*_^NPX(bare)^ for the indivdual case (superscript “NPX” or “bare”) is defined as

13 C̅MNPX(bare)=limT→∞⁡1T∫0T|Im[C0*(t)CM(t)]|dt

with *T* being the total time of simulation, and the coefficients refer to a bare-molecule or a molecule+NP dynamics. In practice, eq 13 is approximated by a sum on the *N*_*s*_ time snapshots {*t*_*i*_} of the discretized dynamics

14 C̅MNPX(bare)≈1Ns∑iNs|Im[C0*(ti)CM(ti)]|

### 2.4 ΔPDOS

Analysis of the electron dynamics is performed using the time-dependent projected density of states (PDOS­(*t*,ε)),^59^ defined as the expectation of the value of the number operator with respect to the wave function |ψ­(*t*)⟩. Specifically, we compute the differential PDOS at time *t* (ΔPDOS), which refers to the initial condition at *t* = 0

15 ΔPDOS(t,ε)=−∑ioccwiRe[∑M,LCL*(t)CM(t)∑avirdi,La*di,Ma]Fη(ε−εi)+∑avirwaRe[∑M,LCL*(t)CM(t)∑ioccdi,La*di,Ma]Fη(ε−εi)

In eq 15, *d*_*i*,*M*_^*a*^ (*d*_*i*,*L*_^*a*^) are the linear coefficients of the expansion for state |*M*⟩ (⟨*L*|) within a singly excited ansatz, and *F*_η_ is a Lorentzian function centered on the molecular-orbital energies ε_*i*_, with width η, used to obtain a smooth profile. The factor *w*_*i*_ denote Mulliken weights; further details are reported in ref 59.

The quantity ΔPDOS provides the time evolution of the molecular-orbital occupations relative to the density of states at time *t* = 0, i.e., the ground-state reference. Equation 10 applies to both the isolated molecule and the molecule+NP system.

## 3 Computational Details

The molecular structure of Chlb is based on a modified Mg-porphyrin in which the phytol tail (C_20_H_39_) is replaced by a hydrogen atom. The ground-state geometry of this structure is optimized at DFT/B3LYP level of theory with a triple-ζ plus polarization (TZP) basis set. Cartesian coordinates of the optimized structure are given in Table S1 of the Supporting Information (SI). Excited-state calculations have been carried out using TDDFT within the Tamm-Dancoff approximation (TDA). All the simulations were performed via the Amsterdam Density Functional (ADF) engine of the Amsterdam Modeling Suite (AMS) software.^66^

The classical gold nanosphere with a radius of 5 nm was modeled with the TDPlas code.^56^ The experimental dielectric function of gold^67^ is fitted by a number of Drude-Lorentz models.^68^ The NP surface is discretized by *N*_*T*_ = 2800 triangular tesserae, and the plasmonic frequency is at 2.5 eV (Figure S1 of SI).

The propagation of the time-dependent wave function is performed using the WaveT package.^56^ Chlb excitation energies and transition dipole moments, together with the NP polarizability obtained from ADF calculations, were used as input parameters for the real-time propagation. To cover the energy range up to 7 eV, the lowest 90 excited states of the molecule are used in the wave function expansion of eq 4. Rotationally averaged absorption spectrum of Chlb is reported in Figure S2 of SI. The presence of low-lying states below 3 eV makes Chlb an optimal candidate to observe plasmonic effects in the ECD spectra.

For both bare molecule and all NPX systems, a 150 fs real-time dynamics simulation was conducted with a time step δt of 1.2 as. The excitation pulse was modeled as a linear Gaussian function as shown in eq 7, polarized along two directions parallel to the molecular plane along trans positioned nitrogen atoms marked as N1–N3 (labeled “∥N1–N3”) and N2–N4 (“∥N2–N4”), and a third direction perpendicular to it (“⊥”), as shown in Figure 1. The external field used in these simulations has a peak intensity of 8.7 × 10^3^ W/cm^2^. A value of 400 au for 1/Γ (eq 10) has been applied consistently in all cases.

## 4 Results and Discussion

To systematically explore the relative orientations of the molecule with respect to the NP, three configurations are considered: (1) the molecular plane parallel to the NP surface (NPX-Parallel Orientation, NP-P), (2) the molecular plane perpendicular to the NP surface along the N1–N3 axis (NPX-Perpendicular N1–N3 Orientation, NP–N1-N3), and (3) the molecular plane perpendicular to the NP surface along the N2–N4 axis (NPX-Perpendicular N2–N4 Orientation, NP–N2-N4). Here, *X* denotes the molecule-NP separation, which was set to 1 nm, 3 or 5 nm. In the following, we refer as *bare* to indicate the isolated molecule in gas phase and *NPX* to denote all the coupled systems at a given distance. A schematic overview of these configurations is provided in Figure 1.

When Chlb is oriented parallel to the NP surface, the distance is measured from the central ion (Mg atom), while for the two perpendicular orientations of the molecule the distance is measured from the atom of the porphyrin plane which is the closest to the NP surface. Three molecule-NP separations were considered for each orientation, namely *d* = 1, 3, and 5 nm. Results for the NP-P and NP–N1-N3 configurations are reported in the main text, in Sections 4.1 and 4.2, while the analysis of NP–N2-N4 case is given in Figures S3–S5 of SI.

Throughout the discussion, we do not use the traditional nomenclature for the optical response of chlorophyll molecules, e.g., Soret, *Q*_*x*_ and *Q*_*y*_ bands.

Before addressing the optical response of Chlb and Chlb+NP, we first analyze the effect of the NP on the ground state of Chlb, i.e., how the ground state is polarized by the presence of the NP under field-free (light-off) conditions.

The polarization charges induced by the NP lead to a shift in the ground-state energy of the molecular system even before the application of the external perturbation. This variation, Δ*E*, is defined as the difference between the ground-state energy of the isolated molecule (“Bare”) and that of the molecule in the presence of the NP at a distance *d* = *X* nm

16 $$\Delta E=E_{\mathrm{GS}}^{\mathrm{Bare}}-E_{\mathrm{GS}}^{\mathrm{NPX}}$$

Δ*E* accounts for both electronic and nuclear contributions to the electrostatic term in the ground-state energy.

Figure 2 shows how the ground-state energy of Chlb changes in the presence of the NP at different distances, compared to the bare molecule for the NP-P orientation. When the distance between the two systems is 1 nm, the stabilization of the ground state is 16.74 cal/mol; this value decreases rapidly to 0.28 cal/mol at 3 nm, while at 5 nm the stabilization is minimal, only 0.039 cal/mol.

**Figure 2:**
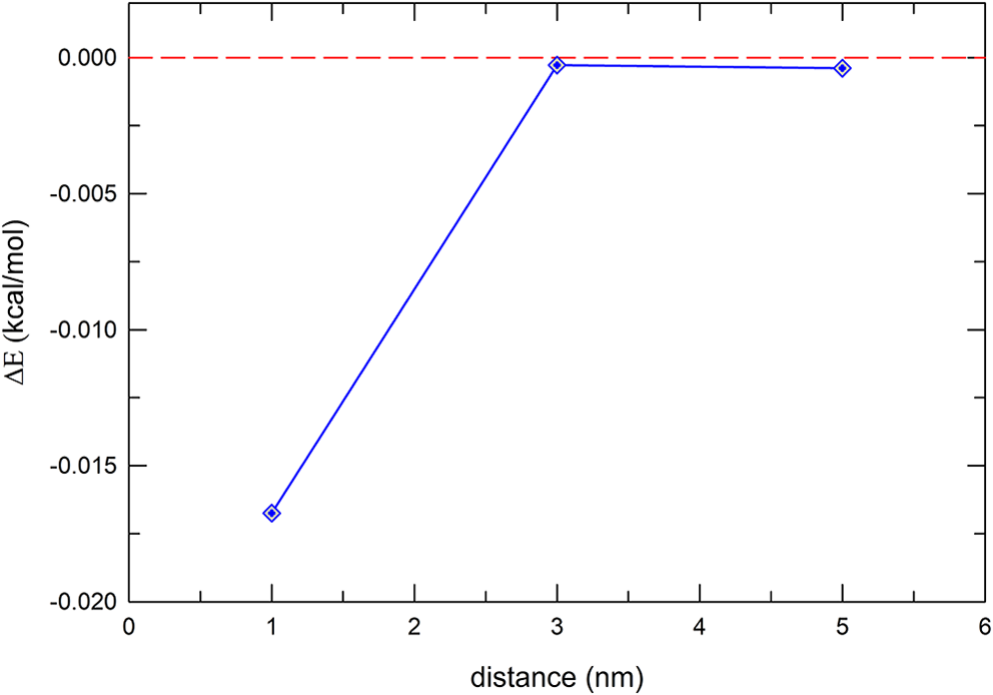
Chlb ground-state energy variation with respect to distance from NP surface for parallel orientation of the molecule with respect to NP surface.

The Δ*E* values for the NP–N1-N3 orientation (very similar to those for NP-NP2-NP4, not reported here) are given in Figure S6 of SI. At 5 nm the stabilization of the ground state is minimal, only 0.055 cal/mol; at 3 nm it increases to 0.35 cal/mol, and at the shortest distance of 1 nm it reaches 12.3 cal/mol.

At 1 nm we observe that parallel orientation of the molecule couples more efficiently with the NP, as expected since in the parallel geometry all atoms of the porphyrin plane are exposed toward the NP, leading to a stronger stabilization.

The ΔPDOS descriptor is a useful tool to analyze the photoinduced time evolution of molecular-orbital occupations, evaluated with respect to the initial condition, i.e., the system (bare Chlb or Chlb+NP) in its electronic ground state. Figures S7–S9 of the SI show the ΔPDOS plots as a function of the orbital energy ε scaled by the HOMO energy ε_HOMO_, for bare Chlb under ∥N1–N3, ∥N2–N4 and ⊥ pulse polarization directions. All results correspond to the time of 1.2 fs, i.e., immediately after the pulse. The occupied orbitals are labeled 1–5, while the virtual orbitals are labeled 1’-5′ (or 1’-7’ in Figure S9). Specifically, the HOMO is designated as 1, followed by HOMO–1 as 2, HOMO–2 as 3, HOMO–5 as 4, and HOMO–6 as 5. For the virtual orbitals, 1’ corresponds to the LUMO, 2’ to LUMO+1, 3′ to LUMO+2, 4’ to LUMO+3, and 5′ to LUMO+4. Additionally, in the perpendicular direction, LUMO+6 and LUMO+12 are labeled as 6’ and 7’, respectively. The corresponding molecular orbitals are shown in Figure S10 of SI.

For the two parallel directions of the pulse (∥N1–N3 in Figure S7 and ∥N2–N4 in Figure S8) ΔPDOS values have the same order of magnitude. The highest contribution in Figure S7 comes from the depopulation of HOMO–1 and HOMO, while LUMO+1 and LUMO+2 are the most populated ones. In Figure S8 we observe a similar depopulation of HOMO and HOMO–1, while the most populated states are LUMO, LUMO+1 and LUMO+2.

When the polarization is perpendicular to the molecular plane as reported in Figure S9, ΔPDOS values are lower by 2 orders of magnitude compared to the in-plane polarization directions of the pulse, which is strictly connected to the lower intensity of the corresponding spectra, as discussed below. Orbital contributions show a different trend, with high-energy virtual orbitals such as LUMO+6 and LUMO+12 being involved.

### 4.1 ECD Spectra for the NP-P Case

We report the ECD spectra of Chlb parallel to the NP (NP-P orientation), and three pulse polarizations, as sketched in Figure 1. For each pulse direction, the ECD spectra of the bare Chlb collected are used as a reference to assess the modifications induced by the presence of the NP.

When the pulse is polarized along the N1–N3 axis (Figure 3), the computed ECD spectra show that all primary peaks exhibit negative intensity across the entire energy range. At a separation of 5 nm, the interaction between the molecule and the NP is almost negligible, leading to a spectral profile that closely matches the bare molecule reference. As the distance decreases to 3 nm, the molecule enters a region where the plasmonic near-field effects become significant. The resulting stronger coupling produces an appreciable enhancement (in absolute value) of the spectral intensity, as the NP-induced electromagnetic field begins to modify the molecular optical response. At the closest distance of 1 nm, the plasmonic coupling is maximal, leading to a substantial amplification of the ECD signal, with peak intensities roughly twice as large as those of the bare molecule. It is also noteworthy that, while the overall intensity increases with decreasing distance, the fundamental features of the spectrum remain largely unchanged. This indicates that the primary electronic transitions of Chlb are not substantially modified by the presence of the NP, and that the dominant effect of the coupling is to act as an amplification mechanism.

**Figure 3:**
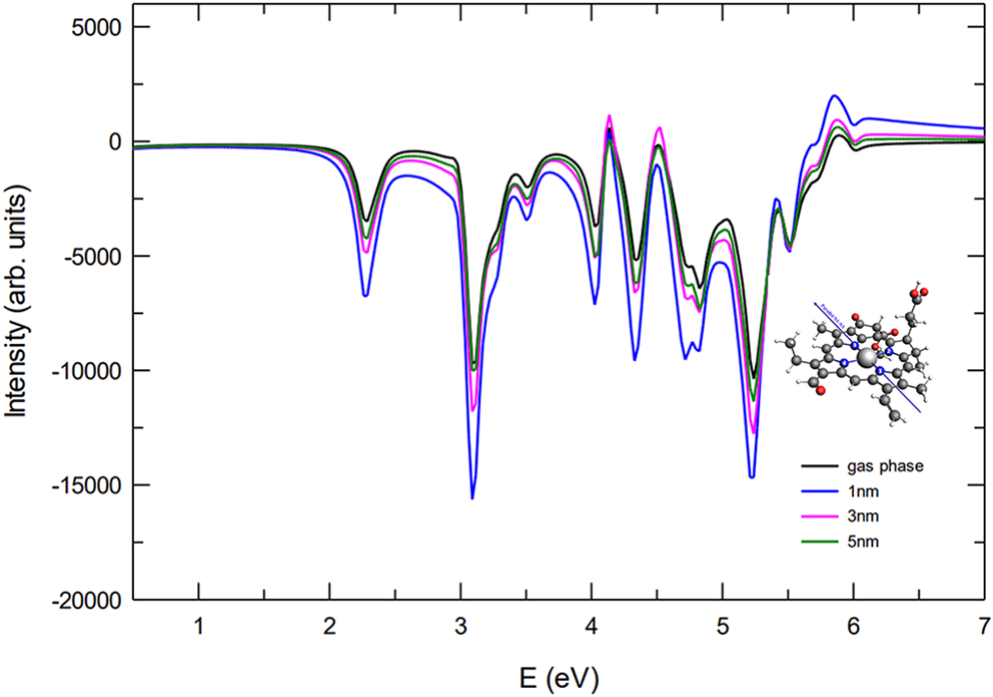
ECD spectra for the Chlb parallel orientation (NP-P) and pulse polarization parallel to the N1–N3 direction. The spectrum of the bare Chlb is reported for comparison.

For the pulse polarized parallel to the N2–N4 axis (Figure 4), the ECD spectra exhibit a distinct intensity distribution pattern, already noticeable in the gas-phase reference. Unlike the case where the pulse is polarized along the N1–N3 axis, here, most peaks display positive intensity, except for the first absorption feature at 2.27 eV and another at 3.2 eV, which retain negative values. When the molecule is coupled with the plasmonic NP, the general trend of distance-dependent enhancement remains evident. At a distance of 5 nm, the spectra closely resemble the gas-phase case, reflecting weak plasmonic interaction. As the molecule approaches the NP at 3 nm, an overall increase in intensity is observed, with a more pronounced enhancement occurring at the lowest separation of 1 nm, where the interaction is strongest. The amplification reaches nearly twice the intensity of the bare-molecule spectra, confirming the role of plasmonic coupling in modifying the chiral optical response. However, it is important to note that the differences between the N1–N3 and N2–N4 polarized spectra persist even in the presence of the NP, although a similar intensity range (in absolute value) is observed in both cases.

**Figure 4:**
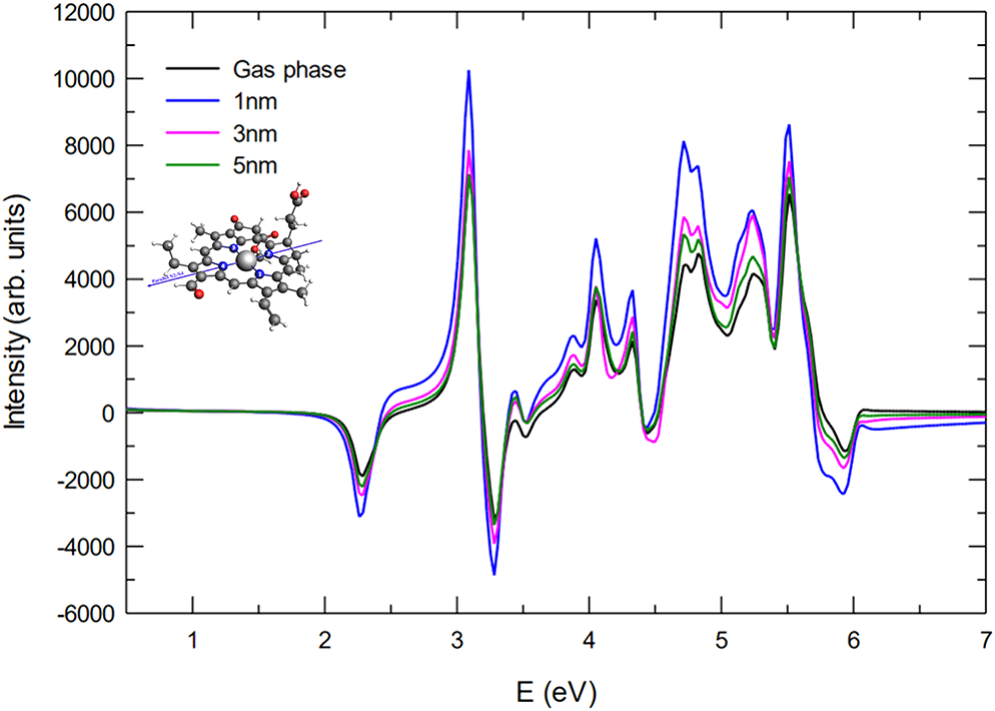
ECD spectra for the parallel orientation (NP-P) and pulse polarization parallel to the N2–N4 direction. The spectrum of the bare Chlb is reported for comparison.

For the case where the pulse is polarized perpendicular to both the molecular plane and the NP surface (Figure 5), the ECD spectra exhibit markedly different features compared to the previous two cases. A striking difference is the much lower intensity observed in the bare-molecule spectrum, reduced by an order of magnitude relative to the parallel polarization cases.

**Figure 5:**
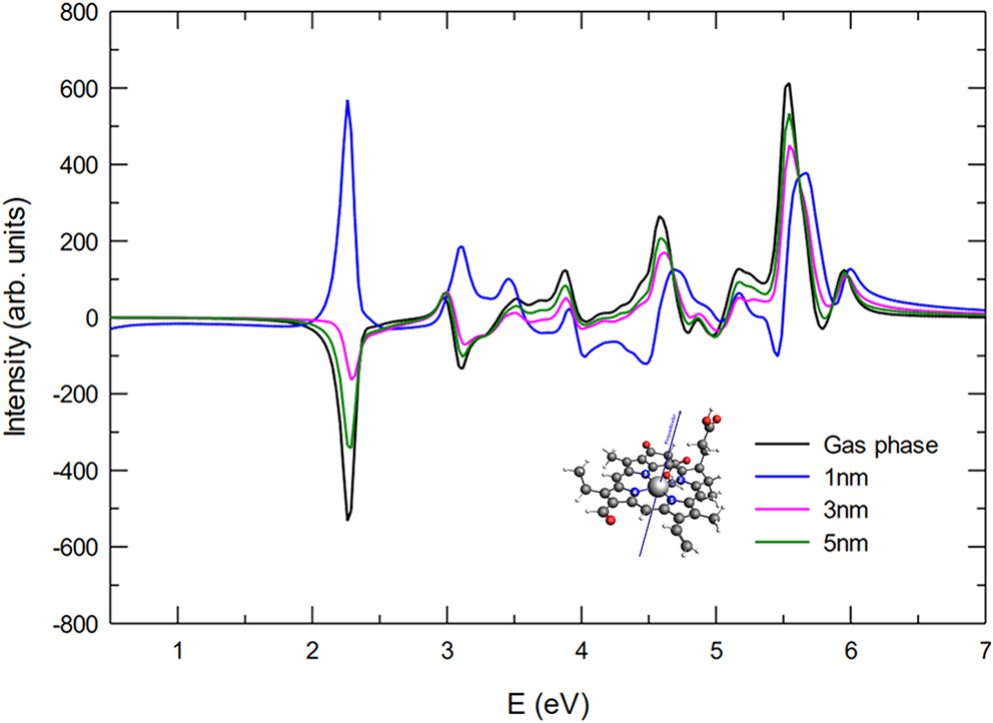
ECD spectra for the NP-P orientation and pulse polarization perpendicular to the Chlb plane. The spectrum of the bare Chlb is reported for comparison.

At a distance of 1 nm, the interaction with the plasmonic field leads to an inversion in the intensity of certain peaks, most notably on 2.27 eV, where the sign flip is accompanied by a strong enhancement in absolute magnitude. A similar, though less pronounced effect, is observed for the peak at 3.1 eV. The trend leading is already present at larger distances of 3 and 5 nm, where a progressive decrease in peak intensity, interpretable as a quenching, is observed compared to the gas-phase spectrum. However, only at the shortest distance of 1 nm does the sign actually flip, indicating a critical threshold where the plasmonic influence overcomes the intrinsic chiral response of the molecule. This phenomenon can be understood in terms of changes in the induced electronic density of the molecule due to the interaction with the plasmonic field.^57^ At close separations, the near-field effects dominate, leading to a redistribution of charge density that alters the chiral response. This interpretation aligns with previous findings in similar systems,^57^ where plasmon-induced modifications to molecular electronic densities have been linked to sign inversions in the ECD spectra.

As mentioned in the Section 12 the coherence descriptor *C̃*_*M*_ is defined in order to get a dynamical “interpretation” of the plasmon-mediated ECD signal. We have considered the first, second and the fourth electronic excitations of Chlb, with corresponding energy values of approximately 2.27 eV, 2.36 and 3.10 eV, respectively. It is worth mentioning that *C̃*_*M*_ does not reproduce the enhancement or quenching factor quantitatively, but only offers a qualitative description of plasmonic effects on the optical response of the molecule.

Table 1 provides the value of *C̃*_*M*_ for those specific excitations of interest, even through the same approach is generally applicable to any excitation. An enhancement for the peak corresponding to the excitation *M* is observed if *C̃*_*M*_ > 1, a quenching corresponds to *C̃*_*M*_ < 1. This simple descriptor is able to capture the qualitative trend in terms of enhancement or quenching of the signal, as one can easily verify by comparing the data in the Table and the ECD spectra commented above: for the two in-plane pulse polarizations (∥N1–N3 and ∥N2–N4) a monotonic trend is seen, with the *C̃*_*M*_ value converging to unity from above, while, by construction, the sign flip can not be detected by *C̃*_*M*_ for the ⊥ pulse polarization, which then shows a nonmonotonic behavior.

**Table 1:** *C̃*_*M*_ Values for the NP-P Orientation

excitation	*d* (nm)	∥ N1–N3	∥ N2–N4	⊥
M = 1	1	1.90	1.78	1.23
	3	1.38	1.35	0.27
	5	1.20	1.19	0.61
M = 2	1	1.85	1.82	1.03
	3	1.33	1.38	0.41
	5	1.16	1.22	0.64
M = 4	1	1.46	1.45	1.10
	3	1.25	1.24	0.60
	5	1.13	1.12	0.82

ΔPDOS for Chlb+NP in the various relative orientations and pulse polarizations (not reported in this work) maintain the same profile and composition for *d* = 3 and 5 nm, while a mixing (also due to known artifacts of Mulliken weights) is found for *d* = 1 nm.

### 4.2 ECD Spectra for the NP–N1-N3 Case

To investigate the influence of molecular orientation relative to the NP surface, we analyzed two configurations where the molecular plane of Chlb is oriented perpendicularly to the NP, i.e., NP–N1-N3 and NP–N2-N4 orientations (Figure 1). In the NP–N1-N3, the axis connecting the nitrogen atoms N1 and N3 of the porphyrin ring is aligned so that it points directly toward the center of the NP.

When the pulse is polarized parallel to the N1–N3 axis of the molecule, the ECD spectra show appreciable changes as a function of the distance *d* with respect to the bare-Chlb case, as reported in Figure 6. At the shortest distance considered (1 nm), a pronounced sign inversion is observed for the first two transitions at 2.27 and 3.1 eV. These transitions are the closest to the NP plasmon resonance (2.5 eV), indicating strong plasmon-induced effects. At an intermediate distance of 3 nm, the plasmonic influence leads to a signal quenching across the entire energy range. At the largest distance of 5 nm, the molecule is sufficiently far from the NP for the plasmonic field to exert only a minor influence. Consequently, the ECD spectra reproduce the bare-Chlb behavior, with only slight reductions in intensity.

**Figure 6:**
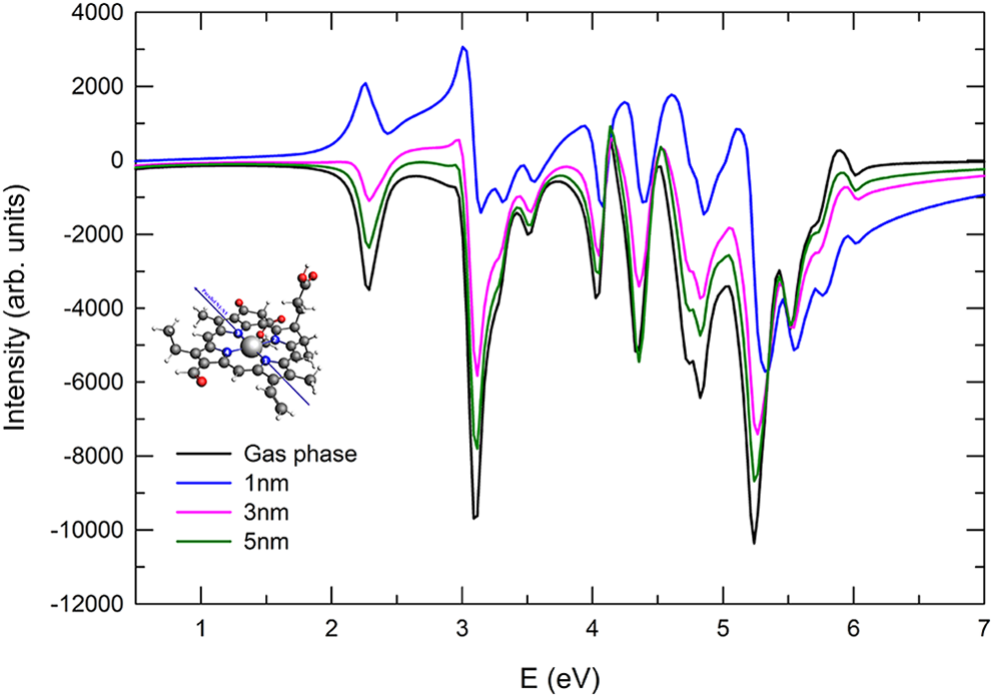
ECD spectra for the NP–N1-N3 orientation and pulse polarization parallel to the N1–N3 direction. The spectrum of the bare Chlb is reported for comparison.

When the polarized pulse is parallel to the N2–N4 axis of the Chlb molecular plane, a different interaction pattern emerges between the molecule and the NP, as shown in the Figure 7. With Chlb at 1 nm from the NP surface, a signal enhancement is observed, especially in the low-energy region, with an intensity approximately two times higher compared to bare-Chlb spectrum. No sign inversion is found in this case. At 3 and 5 nm, the corresponding spectra tend to converge the reference once. As for the NP-P orientation, the intensity range for the two pulse polarizations is similar in absolute value.

**Figure 7:**
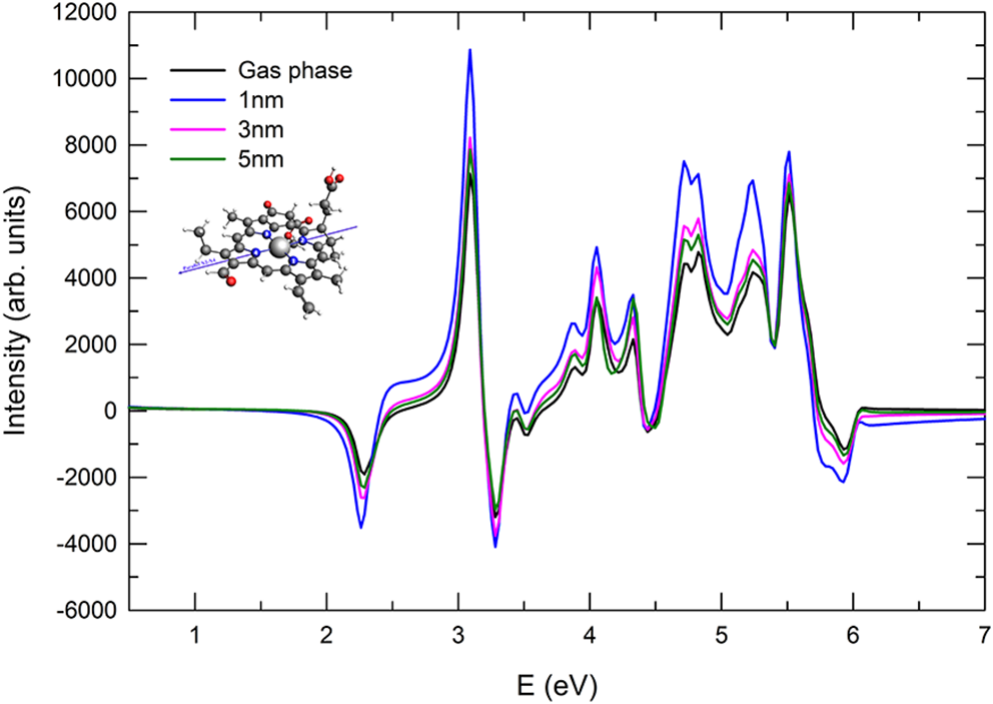
ECD spectra for the NP–N1-N3 orientation and pulse polarization parallel to the N2–N4 direction. The spectrum of the bare Chlb is reported for comparison.

When the polarized pulse is perpendicular to the molecular plane but parallel to the NP surface, the interaction dynamics between the molecule and the NP are markedly different from the previous configurations, as shown in Figure 8. While at a distance of 3 and 5 nm no important changes are observed with respect to the bare-Chlb profile except for a slight enhancement across the entire energy region, the coupling of the two subsystems at a distance of 1 nm instead, gives noticeable modifications in the ECD signal. At 2.36 eV a new peak appears corresponding to the |0⟩ → |2⟩ excitation, which is instead negligible for the isolated Chlb: such excitation is thus strongly enhanced by the presence of the NP.

**Figure 8:**
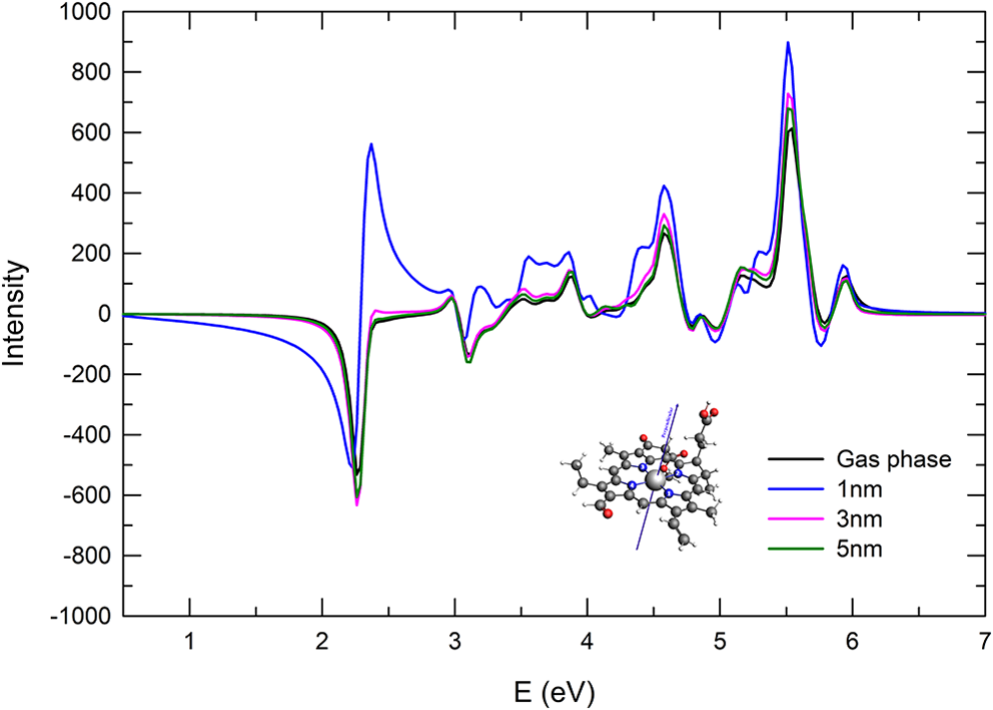
ECD spectra for the N1–N3 perpendicular orientation and pulse polarization perpendicular to the Chl plane. The spectrum of the bare Chlb is reported for comparison.

This specific alignment of the pulse with the NP surface enables the molecule to couple more effectively with the NP plasmon resonance, particularly at transition energies that overlap with the plasmonic peak, which in contrast to the case where the pulse was perpendicular to both the molecular plane and the NP surface, such resonance enhancement was absent. The first peak in the spectra has negative intensity, the new peak induced by NP presence (already present at 3 nm with an intensity smaller by orders of magnitude) shows a rather similar intensity as the first one but with opposite sign, giving a strong intensity enhancement and appearing as a positive Cotton effect.

Table 2 shows the *C̃*_*M*_ values for NP–N1-N3 systems on all the considered distances and the three already mentioned excitations, i.e., M = 1, 2, and 4. When the pulse is polarized along N1–N3 direction we observe a nonmonotonic trend, with the bare-Chlb behavior recovered from below.

**Table 2:** *C̃*_*M*_ Values for the NP–N1-N3 Orientation

excitation	*d* (nm)	∥ N1–N3	∥ N2–N4	⊥
M = 1	1	0.50	2.10	1.33
	3	0.42	1.53	1.21
	5	0.68	1.29	1.15
M = 2	1	0.98	1.57	2.19
	3	0.41	1.31	1.28
	5	0.64	1.18	1.13
M = 4	1	0.48	1.35	1.50
	3	0.66	1.17	1.14
	5	0.81	1.09	1.21

For the N2–N4 pulse direction, the same trend as in the NP-P orientation is found, with an enhancement captured by the coherence. When the external pulse lies perpendicularly on molecular plane, the most striking finding is the large *C̃*_*M*_ value for M = 2, which is not observed for the NP-P orientation.

The changes observed in the ECD spectra are the result of a complex combination of several factors: the gap between the plasmonic frequency and molecular excitations, the amplitude of the electric and magnetic transition dipoles, and their coupling with the polarization direction of the pulse. The final result therefore goes beyond a modeling approach, because it takes into account the characteristics of the chiral molecule, as obtained from first-principles, and of the NP.

Analysis of transition dipole moments computed at TDDFT/TDA level of theory can help in getting a deeper insight into the origin of the presented results. Tables S2 and S3 of SI contain the electric and the magnetic transition dipole moments for the first ten excitations of the bare Chlb. In Tables S4 and S5 of SI one finds the electric and magnetic transition dipole moments for the first ten excitations of Chlb in the NP-P orientation, while the electric and magnetic transition dipole moments for the first ten excitations of Chlb in the NP–N1-N3 orientation are given in Tables S6 and S7 of SI. Tables S4–S7 refer to *d* = 1 nm; for larger distances, the observed effect are substantially negligible. All the tables collect the Cartesian components of the dipoles, and their projection along ∥N1–N3, ∥N2–N4 and ⊥ directions.

Changes in the magnitude of individual dipole components on the pulse polarization directions are in general rather small when compared with those of the bare Chlb, indicating that the polarization on the electronic degrees of freedom does not play an appreciable role in the modification of the ECD spectra. In other words, intensity differences on the ECD spectra for NPx systems can not be generally attributed to a change of the magnitude of the dipole moments, but depend on plasmonic effects, as discussed above and in ref 57. A noticeable exception is provided by the magnetic dipole moment of the second excitation (M = 2) of the Chlb in the NP–N1-N3 orientation, when projected onto the ⊥ direction (Figure 1). This projected dipole is four or five times larger than that of the molecule alone or in other orientations. By comparing the magnetic transition dipole moment for the |0⟩ → |2⟩ excitation of the bare molecule (Table S3 of SI) and of the Chlb+NP at 1 nm in the NP–N1-N3 configuration (Table S7 of SI), one observes that, while the dipole magnitude is not strongly affected by NP polarization, *x* and *z* components of the dipole change sign with the respect to the gas-phase results, i.e., the relative phase in the dipole is modified. This leads to a larger projection of this transition dipole moment onto the direction perpendicular to the molecular plane. This finding explains the appearance of the strong positive peak at around 2.4 eV in Figure 8, and its nature: strictly speaking, it is not due to a plasmonic effect, but to a strong modification of the magnetic response of the second excited state of Chlb. An analysis of the current density, which goes beyond the scope of this work, would allow for a better characterization of this peak in the ECD spectrum.

Instead, electric dipole moments, which are responsible for “selecting” the excited states during the dynamics, are mostly unaffected by the presence of the NP.

## 5 Conclusions

In this work, the ECD response of Chlb in the presence of a gold NP has been investigated by means of a quantum/classical framework. The molecule was described at the quantum level of theory, while the NP was modeled as a polarizable continuum medium. The influence of the NP on the ECD spectrum was explored under different configurations, by varying both the distance between the molecule and the NP surface, and the orientation of the system with respect to the incident electromagnetic field. The results indicate that the presence of the NP can induce a pronounced modulation of the ECD signal, particularly when the molecule is located in proximity to the NP, i.e., at 1 nm. In certain geometries, an enhancement of the ECD response was observed, whereas in other cases a sign inversion of the signal occurred. These effects are strongly dependent on the distance and relative orientation, highlighting the importance of the spatial arrangement in plasmon-assisted spectroscopies. Plasmonic effects are dynamical in nature, and have been seen to modify the time-dependent coefficients, thus enhancing or quenching the ECD signal. The presence of the NP does not alter significantly energies and transition dipole moments, excepting the magnetic transition dipole moment for the second excitation of the Chlb in the NP–N1-N3 orientation, when projected onto the perpendicular direction. Instead, the time-dependent plasmonic induced field plays the fundamental role during the dynamics of modifying populations and coherences, as observed by our coherence-based descriptor, and consequently the ECD response.

To gain further insight into the observed spectral modifications, a detailed analysis of the variation of the ΔPDOS variation was carried out. This allowed us to trace the redistribution of electronic density during the dynamics and to identify the contribution of specific molecular orbitals. Moreover, a coherence descriptor was employed to monitor how quantum coherence between electronic states is modulated by the plasmonic environment. This analysis revealed the impact of coherence on the emergence of plasmon-induced spectral features and provided a consistent interpretation of the observed amplification of the ECD signal.

Beyond the assumptions of our approach, including of the magnetic response of the NP would allow peaks specific to the NP to be observed in the spectrum of the composite system and, in general, is needed for chiral NPs.

## Supplementary Material



## Notes

Chlb Cartesian coordinates used in this work; absorption spectrum of Au NP and bare Chlb; ECD spectra for the N2–N4 perpendicular orientation; ground-state stabilization for the NP–N1-N3 orientation; ΔPDOS for bare Chlb and molecular orbitals; transition dipole moments for bare Chlb and Chlb+NP systems (PDF)
