# Supplementary material for: Simulating the Electronic Circular Dichroism of Chlorophyll b in the Presence of a Gold Nanosphere
Source: J Phys Chem B. 2025 Dec 16;130(1):195–204. doi: 10.1021/acs.jpcb.5c06445 (PMC12794181; doi:10.1021/acs.jpcb.5c06445)
Supplement: Supplementary file 1 [file jp5c06445_si_001.pdf]

Supporting Information for "Simulating the Electronic Circular Dichroism of Chlorophyll b in Presence of a Gold Nanosphere"

Rilinda Plakaj,† Leonardo Biancorosso,† Eleonora Luppi,*,‡ and Emanuele Coccia*,†

†*Dipartimento di Scienze Chimiche e Farmaceutiche, Università di Trieste, via L. Giorgieri 1, 34127, Trieste, Italy*

‡*Laboratoire de Chimie Théorique, Sorbonne Université, CNRS, Paris, F-75005, France*

E-mail: eleonora.luppi@sorbonne-universite.fr; ecoccia@units.it

Table S1: Cartesian coordinates of Chlb.

Atom	x	y	z
C	0.06500	-16.77176	4.85825
C	0.73181	-16.24403	6.03661
N	1.99261	-15.85166	5.72557
C	2.18588	-16.10424	4.38420
C	0.97148	-16.69694	3.83116
C	0.16189	-16.16182	7.31688
C	0.72468	-15.65251	8.47583
N	1.95404	-15.10101	8.59561
C	2.13606	-14.62685	9.87515
C	0.84840	-14.71943	10.67385
C	0.01586	-15.70243	9.81867
C	3.33302	-14.10122	10.29423
C	3.72530	-13.56176	11.67285

Continued on next page

Table S1 (continued)

Atom	X	Y	Z
C	5.21910	-13.06081	11.50157
C	5.58249	-13.35387	10.11789
C	4.47132	-13.95353	9.47371
C	6.59225	-13.24848	9.17732
C	6.02781	-13.80338	7.96289
N	4.71974	-14.22530	8.19569
C	3.60954	-14.60271	12.77646
O	2.76511	-14.59704	13.63839
O	5.85469	-12.53477	12.38677
Mg	3.35340	-15.05076	6.97798
N	4.75360	-14.91602	5.45633
C	4.54147	-15.28316	4.17046
C	5.72644	-15.00918	3.38650
C	6.66612	-14.46954	4.25439
C	6.03747	-14.41337	5.54389
C	6.62455	-13.90959	6.71395
C	7.96251	-12.69080	9.35615
C	0.02922	-17.12944	10.38552
C	0.17427	-13.34396	10.86665
C	-0.03279	-12.56246	9.57290
C	-0.77219	-11.26474	9.78049
O	-1.20188	-10.84783	10.82540
C	3.34427	-15.85177	3.68111
C	8.03906	-13.96349	3.93301
C	8.05561	-12.46379	3.59637
C	0.81803	-17.08327	2.43509
C	0.11705	-18.12638	1.98812
C	-1.35285	-17.23415	4.81375
O	-0.91017	-10.59213	8.60553
H	-1.39393	-9.77179	8.81663
O	4.55497	-15.55004	12.64443
C	4.53531	-16.58759	13.64643
H	-0.84071	-16.55455	7.41815
H	3.36188	-16.10941	2.63278
H	7.64302	-13.55564	6.62907
H	1.03490	-15.12751	11.66911
H	-1.02120	-15.36852	9.72360
H	-0.50077	-17.82028	9.72893

Continued on next page

Table S1 (continued)

Atom	X	Y	Z
H	1.05522	-17.48690	10.49297
H	-0.44864	-17.15253	11.36638
H	0.76533	-12.74307	11.56005
H	-0.78940	-13.50110	11.35779
H	-0.59555	-13.13490	8.82952
H	0.92142	-12.32149	9.09536
H	-1.98953	-16.60747	5.43991
H	-1.73908	-17.18943	3.79594
H	-1.45588	-18.26462	5.16466
H	8.45540	-14.52881	3.09700
H	8.70486	-14.15756	4.77796
H	9.07218	-12.12852	3.38535
H	7.43720	-12.25725	2.72173
H	7.66300	-11.87226	4.42445
H	8.14933	-11.86770	8.66317
H	8.08721	-12.31732	10.37059
H	8.72313	-13.45418	9.17833
H	3.12225	-12.70706	11.98557
H	1.34296	-16.46778	1.71100
H	0.05709	-18.33796	0.92916
H	-0.38728	-18.81071	2.65602
H	5.35479	-17.24960	13.38985
H	4.68209	-16.15633	14.63330
H	3.58675	-17.11815	13.62183
C	5.91153	-15.22358	1.95851
O	5.06886	-15.63980	1.18142
H	6.91917	-14.96993	1.58188

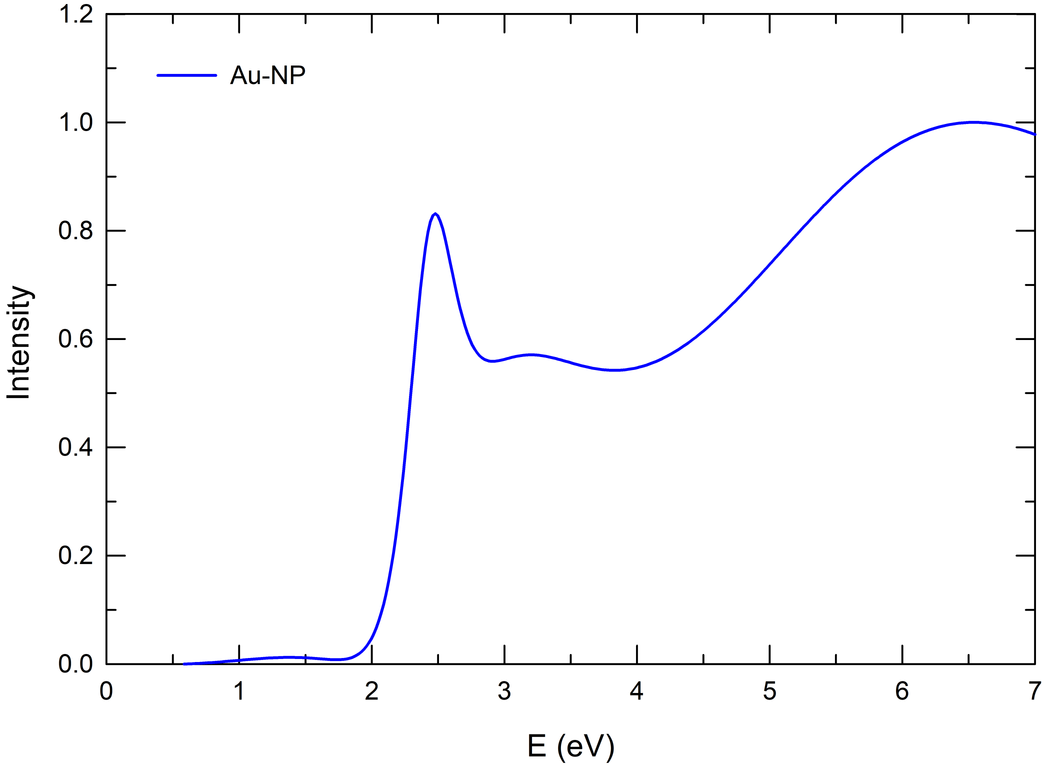

Figure S1: Absorption spectrum of the Au NP.

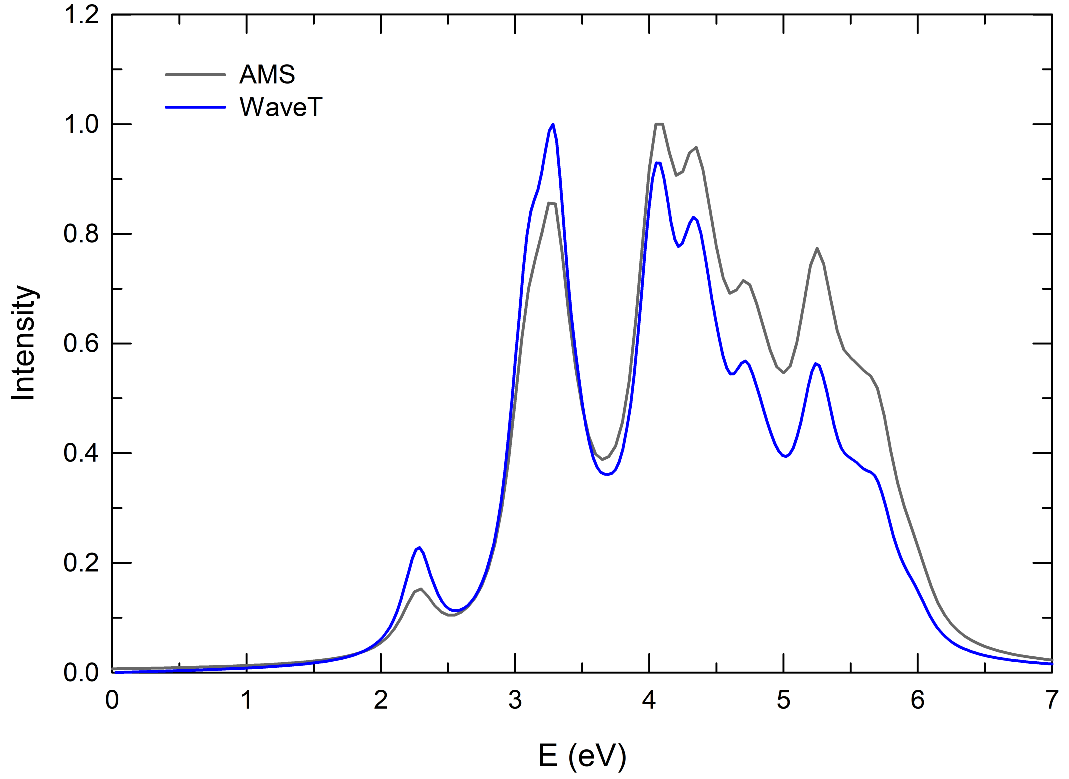

Figure S2: Rotationally-averaged absorption spectrum of the bare Chlb, obtained by a frequency- (AMS) or time-domain (WaveT) calculation.

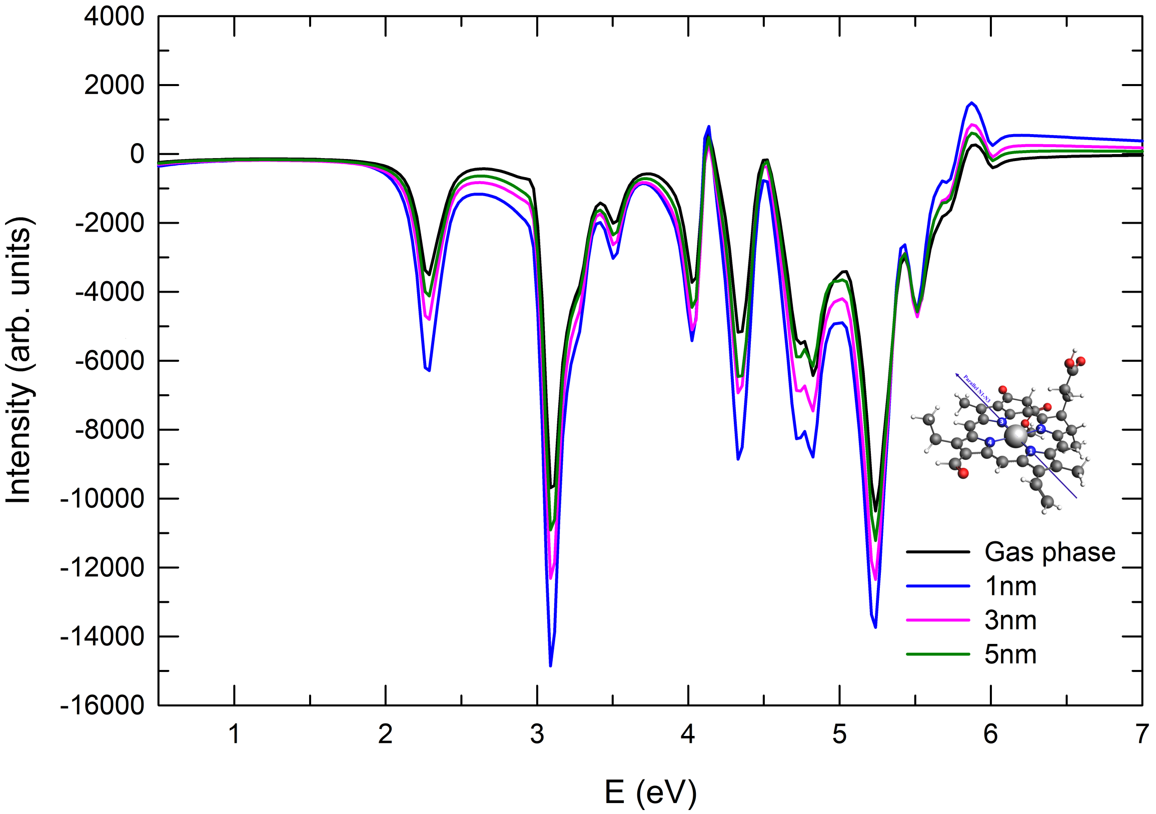

Figure S3: ECD spectra for the N2-N4 perpendicular orientation and pulse polarization parallel to the N1-N3 direction.

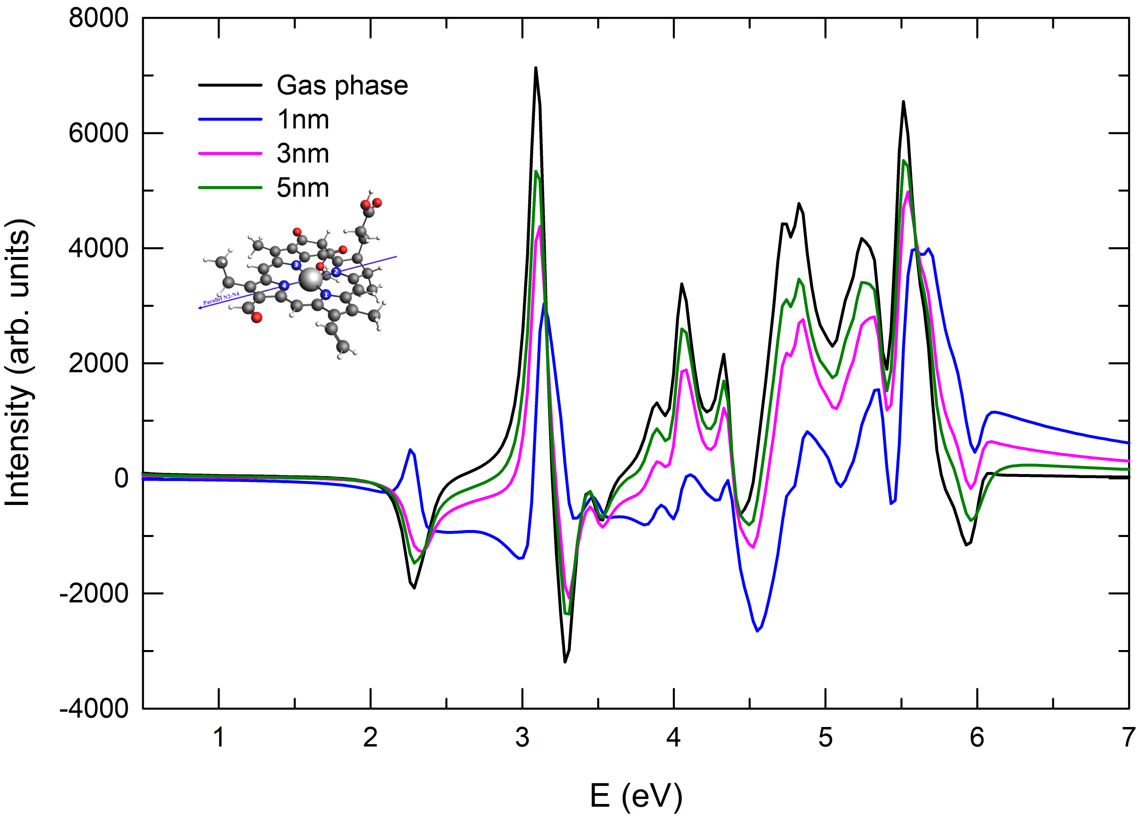

Figure S4: ECD spectra for the N2-N4 perpendicular orientation and pulse polarization parallel to the N2-N4 direction.

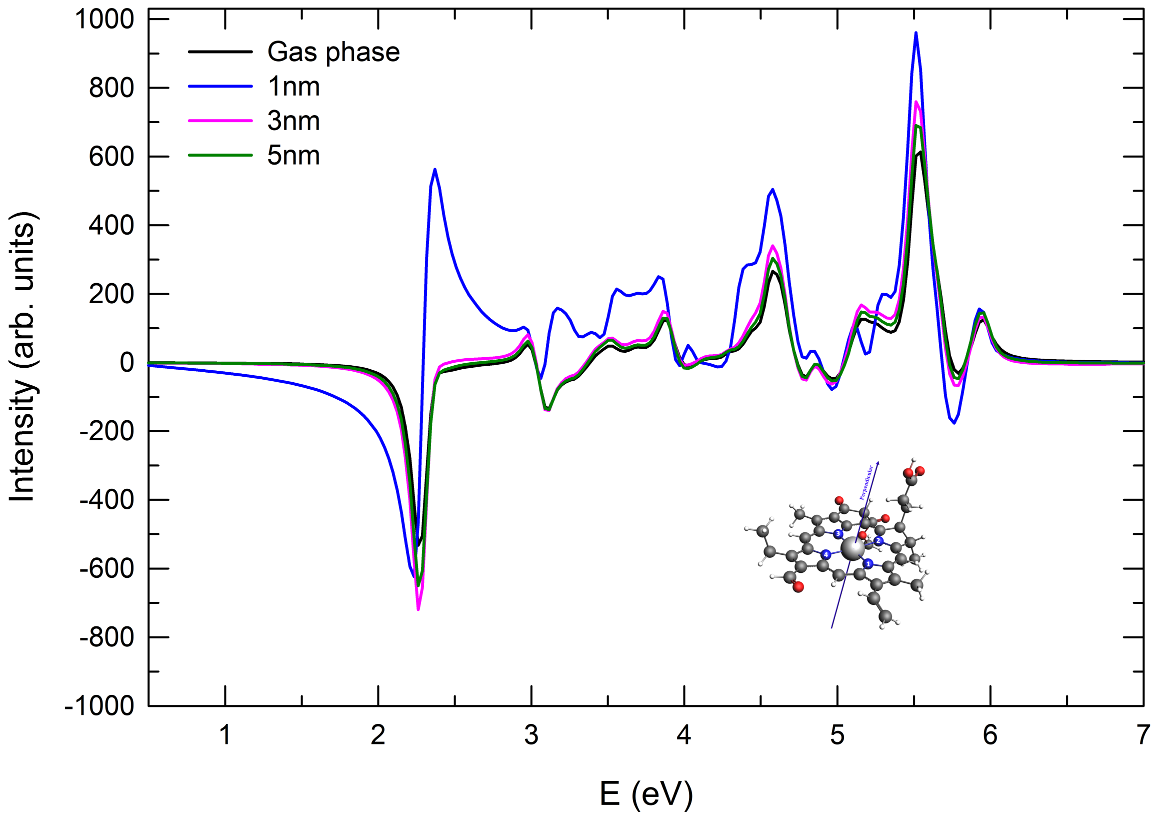

Figure S5: ECD spectra for the N2-N4 perpendicular orientation and pulse polarization perpendicular to the molecular plane.

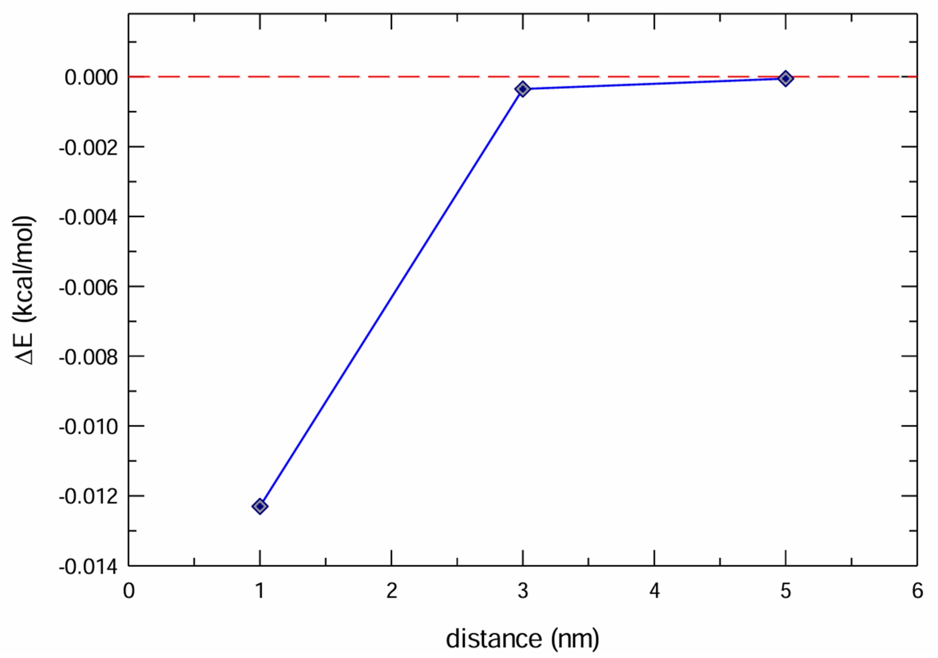

Figure S6: Chlb ground-state energy variation with respect to distance from NP surface for perpendicular N1-N3 orientation of the molecule with respect to NP surface.

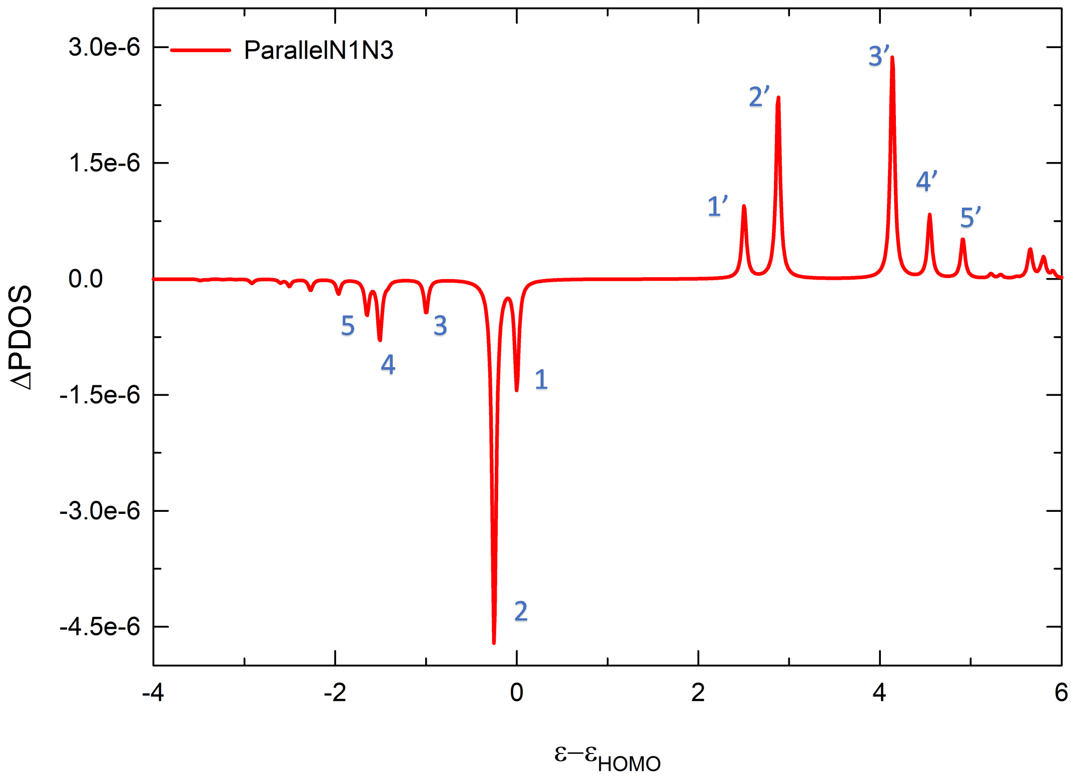

Figure S7: ΔPDOS of the bare molecule for the N1-N3 polarization direction of the pulse at a time step of 1.2 fs.

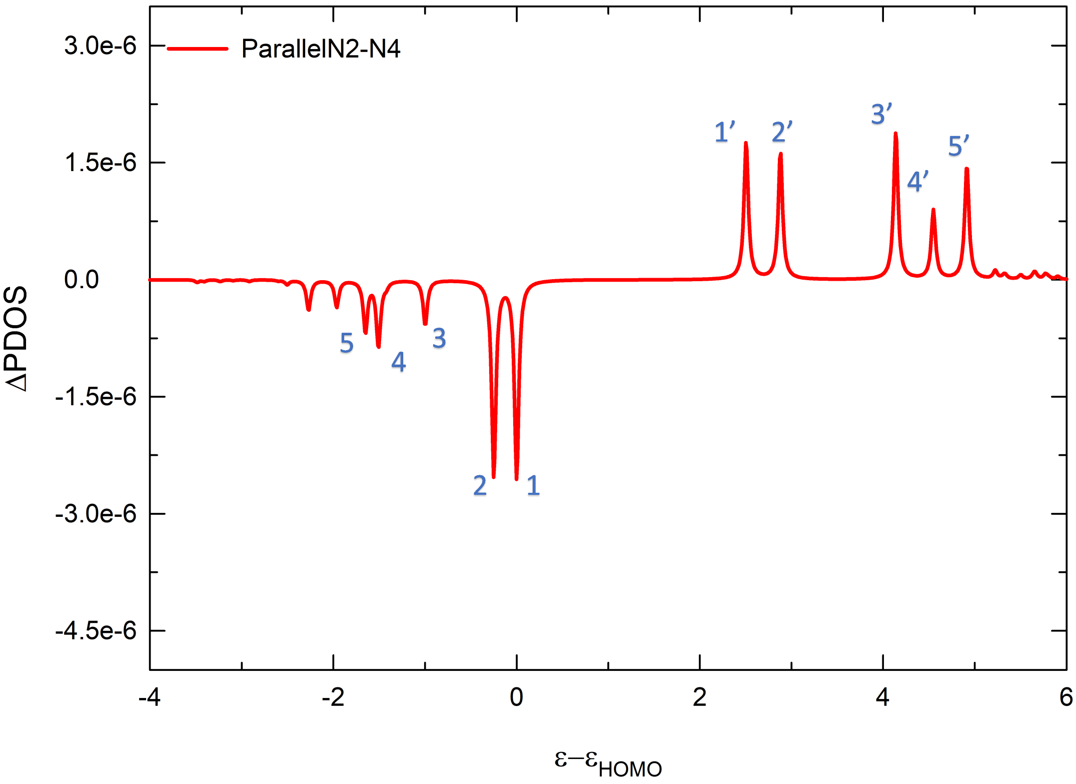

Figure S8: ΔPDOS analysis of the bare molecule for the N2-N4 polarization direction of the pulse at a time step of 1.2 fs.

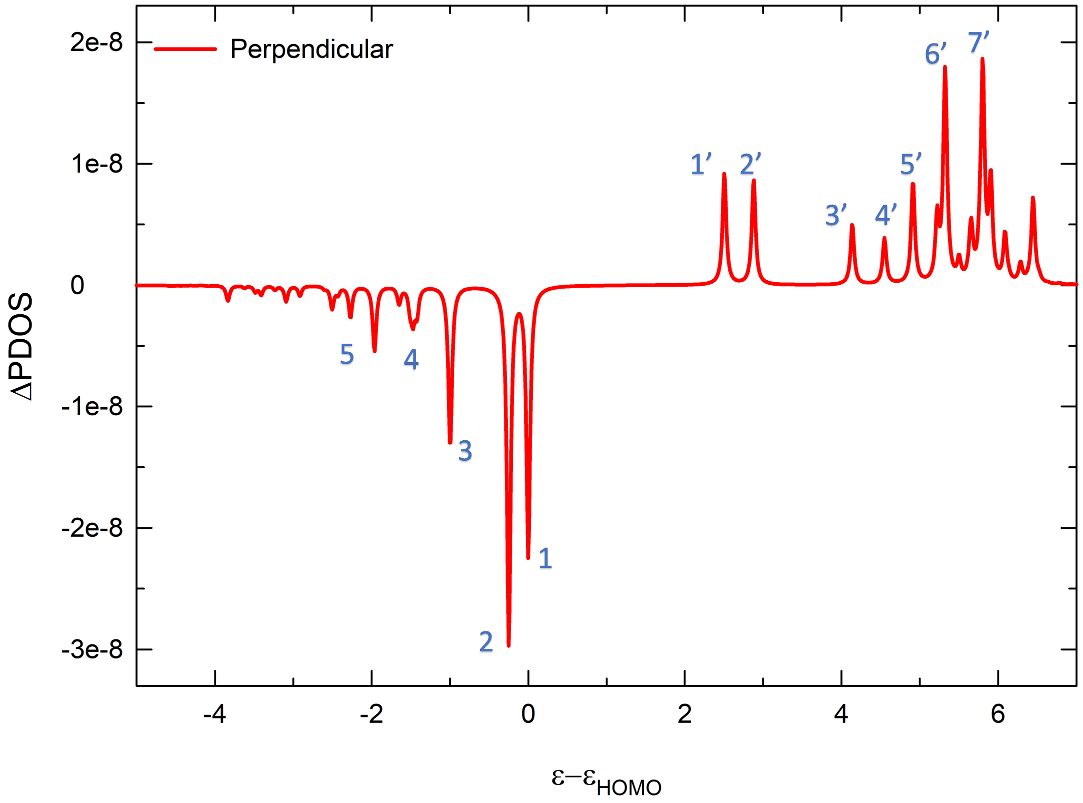

Figure S9: ΔPDOS analysis of the bare molecule for a pulse polarized perpendicularly to the Chlb plane, at a time step of 1.2 fs.

Label	Molecular Orbitals		ε (eV)	ε- ε_{HOMO} (eV)
**5**	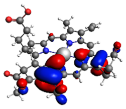	HOMO-6	-7.37	-2.05
**4**	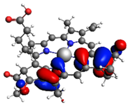	HOMO-5	-7.22	-1.90
**3**	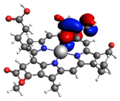	HOMO-2	-6.71	1.39
**2**	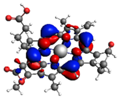	HOMO-1	-5.96	-0.64
**1**	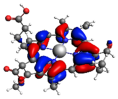	HOMO	-5.32	0.00
**1’**	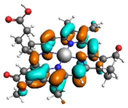	LUMO	-3.22	2.10
**2’**	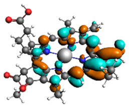	LUMO+1	-2.84	2.48
**3’**	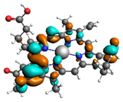	LUMO+2	-1.57	3.75
**4’**	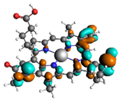	LUMO+3	-1.16	4.16
**5’**	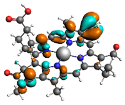	LUMO+4	-0.81	4.51
**6’**	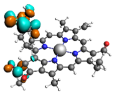	LUMO+6	-0.39	4.93
**7’**	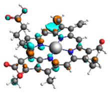	LUMO+12	0.09	5.23

Figure S10: Selected Chlb molecular orbitals.

Transition dipole moments

Tables S3 and S2 provide the magnetic and electric components of the transition dipole moments for the bare molecule, listing excitation energies ΔE and the x, y, and z components of the transition dipole moments, with their projections along the parallel and perpendicular directions for both magnetic and electric transition dipole moments. For clarity, only the first 10 lowest-energy transitions from the ground state to excited states are presented, even though the simulations encompass the full transition dipole matrices.

The first ECD peak, observed at 2.28 eV, is characterized by different one-electron transitions, with the most significant contribution of 61.43% arising from the HOMO $\rightarrow$ LUMO. Additionally, 29.68% of the excitation stems from the HOMO-1 $\rightarrow$ LUMO+1 transition, while minor contributions come from the HOMO $\rightarrow$ LUMO+1 and HOMO-1 $\rightarrow$ LUMO transitions, accounting for 2.87% and 1.59%, respectively. It is worth mentioning that such analysis refer to the rotationally-averaged spectrum, however it cans still useful to understand the transition composition.

For all three polarization directions of the incident pulse, the second peak consistently appears at 3.1 eV, corresponding to the excitation from the ground state to the fourth excited state. Unlike the first peak, this excitation exhibits a more complex orbital contribution. The dominant transition, HOMO-1 $\rightarrow$ LUMO+1, accounts for 38.64%, while HOMO $\rightarrow$ LUMO contributes 13.77 %. Additional contributions arise from the HOMO-1 $\rightarrow$ LUMO+2 transition at 12.5% and the HOMO $\rightarrow$ LUMO+1 transition at 8.86%. The more distributed nature of these contributions indicates increased mixing of electronic states, characteristic of higher-energy excitations.

Table S2: Electric transition dipole moments for the first 10 excitations of the bare Chlb. ΔE in eV and dipoles in atomic units.

Excitation	ΔE	$\vec{\mu}(x)$	$\vec{\mu}(y)$	$\vec{\mu}(z)$	$\vec{\mu}(\parallel$ N1-N3)	$\vec{\mu}(\parallel$ N2-N4)	$\vec{\mu}(\perp)$
$\|0\rangle \rightarrow \|1\rangle$	2.28	-0.96	-0.69	-1.09	-1.60	0.17	0.38
$\|0\rangle \rightarrow \|2\rangle$	2.36	0.14	0.10	0.28	0.31	-0.12	-0.15
$\|0\rangle \rightarrow \|3\rangle$	2.99	0.79	0.49	0.51	1.04	0.15	-0.05
$\|0\rangle \rightarrow \|4\rangle$	3.10	2.26	1.00	0.55	2.27	1.10	0.38
$\|0\rangle \rightarrow \|5\rangle$	3.21	-0.16	-0.28	-0.69	-0.65	0.41	0.39
$\|0\rangle \rightarrow \|6\rangle$	3.29	-0.97	0.34	2.94	1.29	-2.83	-2.26
$\|0\rangle \rightarrow \|7\rangle$	3.35	0.04	0.07	0.08	0.10	-0.04	-0.03
$\|0\rangle \rightarrow \|8\rangle$	3.44	-0.01	-0.31	-1.07	-0.79	0.79	0.68
$\|0\rangle \rightarrow \|9\rangle$	3.45	0.04	0.06	0.13	0.14	-0.07	-0.07
$\|0\rangle \rightarrow \|10\rangle$	3.51	0.01	0.22	0.85	0.61	-0.62	-0.55

Table S3: Magnetic transition dipole moments for the first 10 excitations of the bare Chlb. ΔE in eV and dipoles in atomic units.

Excitation	ΔE	$\vec{m}(x)$	$\vec{m}(y)$	$\vec{m}(z)$	$\vec{m}(\parallel$ N1-N3)	$\vec{m}(\parallel$ N2-N4)	$\vec{m}(\perp)$
$\|0\rangle \rightarrow \|1\rangle$	2.28	-2.28	2.34	1.64	0.41	-2.74	-0.64
$\|0\rangle \rightarrow \|2\rangle$	2.36	0.96	-1.50	-1.09	-0.62	1.45	0.35
$\|0\rangle \rightarrow \|3\rangle$	2.99	0.29	0.58	0.14	0.52	0.08	0.18
$\|0\rangle \rightarrow \|4\rangle$	3.10	0.85	-1.43	-2.03	-1.25	2.08	1.09
$\|0\rangle \rightarrow \|5\rangle$	3.21	-0.12	0.41	1.01	0.71	-0.83	-0.62
$\|0\rangle \rightarrow \|6\rangle$	3.29	-0.09	-0.08	-0.64	-0.48	0.42	0.44
$\|0\rangle \rightarrow \|7\rangle$	3.35	0.29	0.10	0.34	0.45	-0.06	-0.17
$\|0\rangle \rightarrow \|8\rangle$	3.44	0.26	-0.35	-0.10	-0.03	0.25	-0.02
$\|0\rangle \rightarrow \|9\rangle$	3.45	0.15	-0.17	-0.10	-0.03	0.17	0.03
$\|0\rangle \rightarrow \|10\rangle$	3.51	0.16	-1.30	-0.63	-0.80	0.58	-0.04

Table S4: Electric transition dipole moments for the first 10 excitations for NP-P and $d = 1$ nm. ΔE in eV and dipoles in atomic units.

Excitation	ΔE	$\vec{\mu}_x$	$\vec{\mu}_y$	$\vec{\mu}_z$	$\vec{\mu}(\parallel$ N1-N3)	$\vec{\mu}(\parallel$ N2-N4)	$\vec{\mu}(\perp)$
$\lvert 0\rangle \to \lvert 1\rangle$	2.28	0.96	0.69	1.08	1.59	-0.17	-0.38
$\lvert 0\rangle \to \lvert 2\rangle$	2.36	0.14	0.10	0.28	0.31	-0.12	-0.15
$\lvert 0\rangle \to \lvert 3\rangle$	2.99	0.80	0.50	0.51	1.06	0.15	-0.05
$\lvert 0\rangle \to \lvert 4\rangle$	3.10	-2.26	-1.00	-0.55	-2.28	-1.10	-0.38
$\lvert 0\rangle \to \lvert 5\rangle$	3.21	-0.15	-0.27	-0.70	-0.64	0.42	0.39
$\lvert 0\rangle \to \lvert 6\rangle$	3.29	-0.97	0.33	2.94	1.28	-2.84	-2.26
$\lvert 0\rangle \to \lvert 7\rangle$	3.35	0.03	0.07	0.08	0.10	-0.04	-0.03
$\lvert 0\rangle \to \lvert 8\rangle$	3.44	-0.01	-0.31	-1.08	-0.79	0.80	0.69
$\lvert 0\rangle \to \lvert 9\rangle$	3.45	0.04	0.07	0.15	0.15	-0.08	-0.07
$\lvert 0\rangle \to \lvert 10\rangle$	3.51	0.00	-0.22	-0.85	-0.61	0.63	0.55

Table S5: Magnetic transition dipole moments for the first 10 Chlb excitations in the NP-P orientation at 1 nm. ΔE in eV and dipoles in atomic units.

Excitation	ΔE	$\vec{m}(x)$	$\vec{m}(y)$	$\vec{m}(z)$	$\vec{m}(\parallel$ N1-N3)	$\vec{m}(\parallel$ N2-N4)	$\vec{m}(\perp)$
$\lvert 0\rangle \to \lvert 1\rangle$	2.28	2.31	-2.32	-1.63	-0.37	2.75	0.65
$\lvert 0\rangle \to \lvert 2\rangle$	2.36	0.99	-1.54	-1.17	-0.67	1.53	0.41
$\lvert 0\rangle \to \lvert 3\rangle$	2.99	0.12	0.63	0.26	0.50	-0.11	0.09
$\lvert 0\rangle \to \lvert 4\rangle$	3.10	-0.80	1.37	1.87	1.16	-1.93	-0.98
$\lvert 0\rangle \to \lvert 5\rangle$	3.21	-0.22	0.33	0.92	0.55	-0.83	-0.60
$\lvert 0\rangle \to \lvert 6\rangle$	3.29	-0.11	-0.11	-0.68	-0.53	0.43	0.45
$\lvert 0\rangle \to \lvert 7\rangle$	3.35	0.31	0.07	0.26	0.40	0.01	-0.12
$\lvert 0\rangle \to \lvert 8\rangle$	3.44	0.38	-0.38	-0.17	0.00	0.38	0.03
$\lvert 0\rangle \to \lvert 9\rangle$	3.45	0.14	-0.18	-0.10	-0.04	0.17	0.03
$\lvert 0\rangle \to \lvert 10\rangle$	3.51	-0.07	0.13	0.62	0.39	-0.51	-0.43

Table S6: Electric transition dipole moments for the first 10 excitations for NP-N1-N3 and $d = 1$ nm. ΔE in eV and dipoles in in atomic units.

Excitation	ΔE	$\vec{\mu}_x$	$\vec{\mu}_y$	$\vec{\mu}_z$	$\vec{\mu}(\parallel$ N1-N3)	$\vec{\mu}(\parallel$ N2-N4)	$\vec{\mu}(\perp)$
$\|0\rangle \rightarrow \|1\rangle$	2.28	0.96	0.69	1.09	1.60	-0.17	-0.38
$\|0\rangle \rightarrow \|2\rangle$	2.36	-0.14	-0.10	-0.28	-0.31	0.12	0.15
$\|0\rangle \rightarrow \|3\rangle$	2.99	-0.77	-0.48	-0.50	-1.02	-0.14	0.05
$\|0\rangle \rightarrow \|4\rangle$	3.10	-2.27	-1.00	-0.54	-2.27	-1.11	-0.39
$\|0\rangle \rightarrow \|5\rangle$	3.21	-0.16	-0.28	-0.71	-0.66	0.43	0.40
$\|0\rangle \rightarrow \|6\rangle$	3.29	-0.97	0.33	2.93	1.28	-2.83	-2.26
$\|0\rangle \rightarrow \|7\rangle$	3.35	-0.03	-0.07	-0.09	-0.10	0.04	0.03
$\|0\rangle \rightarrow \|8\rangle$	3.44	0.00	0.31	1.08	0.79	-0.80	-0.69
$\|0\rangle \rightarrow \|9\rangle$	3.45	-0.04	-0.06	-0.12	-0.13	0.06	0.06
$\|0\rangle \rightarrow \|10\rangle$	3.51	0.02	0.22	0.85	0.62	-0.62	-0.55

Table S7: Magnetic transition dipole moments for the first 10 Chlb excitations in the NP-N1-N3 configuration and the NP at 1 nm. ΔE in eV and dipoles in atomic units.

Excitation	ΔE	$\vec{m}(x)$	$\vec{m}(y)$	$\vec{m}(z)$	$\vec{m}(\parallel$ N1-N3)	$\vec{m}(\parallel$ N2-N4)	$\vec{m}(\perp)$
$\|0\rangle \rightarrow \|1\rangle$	2.28	2.31	-2.34	-1.66	-0.40	2.77	0.66
$\|0\rangle \rightarrow \|2\rangle$	2.36	-1.01	-1.54	1.18	-0.58	-1.55	-1.72
$\|0\rangle \rightarrow \|3\rangle$	2.99	-0.26	-0.58	-0.14	-0.50	-0.07	-0.18
$\|0\rangle \rightarrow \|4\rangle$	3.10	-0.97	1.40	2.06	1.18	-2.18	-1.14
$\|0\rangle \rightarrow \|5\rangle$	3.21	-0.09	0.29	0.77	0.53	-0.63	-0.48
$\|0\rangle \rightarrow \|6\rangle$	3.29	-0.11	-0.11	-0.68	-0.53	0.44	0.46
$\|0\rangle \rightarrow \|7\rangle$	3.35	-0.30	-0.06	-0.25	-0.38	-0.01	0.12
$\|0\rangle \rightarrow \|8\rangle$	3.44	-0.28	0.33	0.08	-0.01	-0.25	0.03
$\|0\rangle \rightarrow \|9\rangle$	3.45	-0.08	0.18	0.10	0.08	-0.13	-0.01
$\|0\rangle \rightarrow \|10\rangle$	3.51	0.02	-1.32	-0.61	-0.89	0.47	-0.09
